# Draft Genome Sequences of 42 *Helicobacter pylori* Isolates from Rural Regions of South India

**DOI:** 10.1128/genomeA.01486-17

**Published:** 2018-02-01

**Authors:** Vignesh Shetty, Binit Lamichhane, Eng-Guan Chua, Mamatha Ballal, Chin-Yen Tay

**Affiliations:** a Enteric Diseases Division, Department of Microbiology, Kasturba Medical College, Manipal University, Manipal, India; b *Helicobacter pylori* Research Laboratory, Marshall Centre for Infectious Diseases Research and Training School of Biomedical Sciences, University of Western Australia, Nedlands, Western Australia, Australia

## Abstract

*Helicobacter pylori* is a successful human gastric pathogen that is associated with the development of gastric cancer. The draft genome sequences of 42 *H. pylori* clinical strains isolated from South Indian rural populations will provide further insights into the evolution and genetic makeup of Indian *H. pylori* strains.

## GENOME ANNOUNCEMENT

*Helicobacter pylori* infection is one of the major public health problems in India. In rural areas, the prevalence of *H. pylori* infection is at least 80%, and peptic ulcer disease is the most common clinical manifestation (1). Interestingly, the incidence of duodenal ulcer disease was reported to be several times greater than that of gastric ulcer disease in South Indian populations (2).

In this report, we present the draft genome sequences of 42 *H. pylori* strains isolated from patients with gastritis and peptic ulcer disease. The gastric biopsy specimens of patients from remote villages in the Karnataka and Kerala states of India were collected at the gastroenterology and general surgery departments in Kasturba Medical College Hospital in Manipal, India. This study was approved by the Manipal University Human Ethics Committee (reference no. IEC301/2014). Bacteria were grown on brain heart infusion agar supplemented with 5% (vol/vol) horse serum, 0.5% (vol/vol) BBL IsoVitaleX enrichment medium (Becton, Dickinson and Company, USA), trimethoprim (5 µg/ml), and vancomycin (6 µg/ml) for 48 h in a humidified 10% CO_2_ environment. Cells were then harvested, and genomic DNA was extracted using the cetyltrimethylammonium bromide (CTAB) DNA isolation technique as described by Stewart and Via (3). Sequencing libraries were prepared using a Nextera XT kit (Illumina, USA), and the genomes were sequenced using the 2 × 250 paired-end sequencing protocol on an Illumina MiSeq instrument. After quality trimming reads using CLC Genomics Workbench 10.1.1, reads were assembled with SPAdes (version 3.10.1) (4).

The average G+C content was 39%, and the average genome size was approximately 1.6 Mbp, consistent with results of previous reports (5–7). Multilocus sequence typing (MLST) analysis using seven housekeeping genes, including *atpA*, *efp*, *mutY*, *ppa*, *trpC*, *ureI*, and* yphC*, further divided these strains into two major *H. pylori* populations, hpAsia2 and hpEurope.

Our collection of *H. pylori* draft genomes from South India will allow the scientific community to identify whether *H. pylori* genetic factors (if present) play a role in disease development, particularly in that of duodenal ulcer disease, and to compare this role to that in closely related strains from the northern regions of India. These genomes will also provide further insights into the population genetic structure and evolution of *H. pylori* populations in India.

### Accession number(s).

All draft genome sequences have been deposited at DDBJ/ENA/GenBank under the accession numbers listed in Table 1.

**TABLE 1:** Metadata and genome accession numbers of draft genome sequences reported in this study

Strain	GenBank accession no.	Diagnosis	Origin in India	No. of contigs >500 bp	Genome size (bp)	GC content (%)	MLST population
KH1	PHMW00000000	Acute follicular gastritis with intestinal metaplasia	Haveri, Karnataka	37	1,655,497	39	hpEurope
KH2	PHMV00000000	Gastric ulcer	Udupi, Karnataka	48	1,605,233	39	hpEurope
KH3	PHMU00000000	Chronic active gastritis	Davangere, Karnataka	45	1,615,471	39	hpEurope
KH4	PHMT00000000	Duodenal ulcer	Udupi, Karnataka	30	1,600,701	39	hpEurope
KH6	PHMS00000000	Chronic active gastritis	Davangere, Karnataka	37	1,541,249	39	hpEurope
KH7	PHMR00000000	Chronic active gastritis	Uttar Kannada, Karnataka	36	1,612,680	39	hpAsia2
KH8	PHMQ00000000	Gastric ulcer	Shimoga, Karnataka	41	1,644,428	39	hpEurope
KH9	PHMP00000000	Duodenal ulcer	Davangere, Karnataka	43	1,601,796	39	hpEurope
KH10	PHMO00000000	Duodenal ulcer	Kannur, Kerala	38	1,632,482	39	hpAsia2
KH11	PHMN00000000	Chronic active gastritis	Shimoga, Karnataka	29	1,611,816	39	hpAsia2
KH12	PHMM00000000	Chronic active follicular gastritis	Chitradurga, Karnataka	25	1,532,769	39	hpEurope
KH13	PHML00000000	Chronic active gastritis	Shimoga, Karnataka	38	1,627,704	39	hpEurope
KH14	PHMK00000000	Chronic active gastritis	Shimoga, Karnataka	42	1,596,229	39	hpAsia2
KH15	PHMJ00000000	Chronic follicular gastritis	Udupi, Karnataka	44	1,605,081	39	hpAsia2
KH16	PHMI00000000	Chronic active gastritis	Udupi, Karnataka	46	1,600,481	39	hpEurope
KH17	PHMH00000000	Chronic active gastritis	Uttar Kannada, Karnataka	39	1,626,136	39	hpAsia2
KH18	PHMG00000000	Chronic active gastritis	Shimoga, Karnataka	55	1,648,474	39	hpEurope
KH19	PHMF00000000	Gastric and duodenal ulcer	Davangere, Karnataka	38	1,656,353	39	hpEurope
KH20	PHME00000000	Duodenal ulcer	Chitradurga, Karnataka	42	1,647,666	39	hpEurope
KH21	PHMD00000000	Duodenal ulcer	Chitradurga, Karnataka	46	1,647,799	39	hpEurope
KH22	PHMC00000000	Duodenal ulcer	Shimoga, Karnataka	40	1,592,933	39	hpAsia2
KH23	PHMB00000000	Duodenal ulcer	Davangere, Karnataka	79	1,658,056	39	hpEurope
KH25	PHMA00000000	Chronic active gastritis	Davangere, Karnataka	37	1,625,764	39	hpAsia2
KH26	PHLZ00000000	Chronic active follicular gastritis	Shimoga, Karnataka	62	1,711,421	39	hpEurope
KH27	PHLY00000000	Chronic active gastritis	Davangere, Karnataka	36	1,635,551	39	hpEurope
KH28	PHLX00000000	Chronic active gastritis	Davangere, Karnataka	31	1,681,866	39	hpEurope
KH29	PHLW00000000	Active follicular gastritis	Uttar Kannada, Karnataka	38	1,604,955	39	hpEurope
KH30	PHLV00000000	Active corporal gastritis	Davangere, Karnataka	33	1,580,425	39	hpEurope
KH31	PHLU00000000	Chronic active follicular gastritis	Kannur, Kerala	34	1,607,977	39	hpAsia2
KH32	PHLT00000000	Chronic active gastritis with intestinal metaplasia	Udupi, Karnataka	34	1,611,904	39	hpAsia2
KH33	PHLS00000000	Gastric and duodenal ulcer	Davangere, Karnataka	34	1,614,424	39	hpEurope
KH34	PHLR00000000	Chronic active gastritis	Kannur, Kerala	32	1,583,464	39	hpAsia2
KH35	PHLQ00000000	Chronic active gastritis	Udupi, Karnataka	45	1,657,309	39	hpEurope
KH36	PHLP00000000	Chronic active gastritis	Chitradurga, Karnataka	41	1,644,595	39	hpEurope
KH37	PHLO00000000	Duodenal ulcer	Shimoga, Karnataka	41	1,623,762	39	hpAsia2
KH38	PHLN00000000	Chronic active gastritis with intestinal metaplasia	Davangere, Karnataka	60	1,623,977	39	hpEurope
KH39	PHLM00000000	Gastric and duodenal ulcer	Kannur, Kerala	34	1,622,171	39	hpAsia2
KH40	PHLL00000000	Chronic active gastritis	Udupi, Karnataka	31	1,591,033	39	hpAsia2
KH41	PHLK00000000	Chronic active gastritis	Chitradurga, Karnataka	46	1,654,320	39	hpEurope
KH43	PHLJ00000000	Chronic active gastritis	Udupi, Karnataka	34	1,627,074	39	hpAsia2
KH44	PHLI00000000	Chronic active follicular gastritis	Kannur, Kerala	38	1,615,682	39	hpEurope
KH45	PHLH00000000	Chronic active gastritis	Udupi, Karnataka	36	1,619,626	39	hpAsia2
